# Prognostic Value of Combined Preoperative Carcinoembryonic Antigen and Prognostic Nutritional Index in Patients With Stage II–III Colon Cancer

**DOI:** 10.3389/fsurg.2021.667154

**Published:** 2021-07-20

**Authors:** Yan-song Xu, Gang Liu, Chang Zhao, Shao-long Lu, Chen-yan Long, Hua-ge Zhong, Yi Chen, Ling-xu Huang, Zheng Liang

**Affiliations:** ^1^Department of Emergency, The First Affiliated Hospital of Guangxi Medical University, Nanning, China; ^2^Department of Gastrointestinal and Anorectal Surgery, Nanning First People's Hospital, Nanning, China; ^3^Department of Colorectal Surgery, The Eighth Hospital of Wuhan, Wuhan, China; ^4^Department of Hepatobiliary Surgery, Guangxi Medical University Cancer Hospital, Nanning, China; ^5^Second Department of General Surgery, Zhuzhou Central Hospital, Zhuzhou, China; ^6^Guangxi Clinical Research Center for Colorectal Cancer, Nanning, China

**Keywords:** colon cancer, tumor marker, nutritional status, prognosis, nomogram

## Abstract

**Background:** Tumor status can affect patient prognosis. Prognostic nutritional index (PNI), as a nutritional indicator, is closely related to the prognosis of cancer. However, few studies have examined the combined prognostic value of CEA and PNI in patients. This study investigated the relationship between CEA/PNI and prognosis of colon cancer patients.

**Methods:** A total of 513 patients with stage II–III colon cancer who underwent curative resection at two medical centers from 2009 to 2019 were included. Clinicopathological factors were assessed and overall survival (OS) was assessed in a cohort of 413 patients. Multivariate analysis was used to identify independent prognostic variables to construct histograms predicting 1-year and 3-year OS. Data from 100 independent patients in the validation group was used to validate the prognostic model.

**Results:** The median OS time was 33.6 months, and mortality was observed in 54 patients. Multivariate analysis revealed that preoperative CEA/PNI, lymph node metastasis, peripheral nerve invasion, operation mode, and postoperative chemotherapy were independent factors for prognosis evaluation and thus were utilized to develop the nomogram. The C-index was 0.788 in the learning set and 0.836 in the validation set. The calibration curves reached favorable consensus among the 1-, 3-year OS prediction and actual observation.

**Conclusion:** The combined use of CEA and PNI is an independent prognostic factor and thus can serve as a basis for a model to predict the prognosis of patients with stage II–III colon cancer.

## Introduction

The latest global cancer data showed that colorectal cancer is among the top three with the highest incidence and mortality (1–3). Colon cancer accounts for approximately 65–70% of colorectal cancer cases (4, 5). Complete surgical resection remains the best treatment for patients with non-metastatic colon cancer. Despite receiving curative-intent treatment, 11.6–33% of patients with stage II–III colon cancer would still develop distant metastases or local recurrence 5 years after surgery (6–8). High preoperative CEA level increases the risk of death by 62% compared with preoperative CEA level in non-metastatic colon cancer (9). Therefore, identifying prognostic factors and individualizing postoperative therapy according to patient classification are necessary. Accumulating evidence indicates that nutritional status is associated with survival outcomes in patients with different cancers. Approximately 50–80% of admitted patients with malignant cancers are malnourished or at high risk for malnutrition (10–12). Therefore, an accurate understanding of the nutritional status of cancer patients is helpful to analyze and improve the prognosis of patients.

Prognostic nutritional index (PNI) was first designed by Buzby et al. in gastrointestinal cancer (13), and its relationship with the prognosis of malignant tumors has been subsequently widely studied (14–17). PNI has attracted extensive attention from clinicians due to its convenience in prognostic assessment (18–20). This index is calculated by the serum albumin and the total number of peripheral blood lymphocytes. Serum albumin, which is synthesized in and secreted from the liver, reflects the host's nutritional status and has a decreased level in malignant cancers (14–17, 21). Lymphocytes can recognize and eliminate tumor cells; the reduced numbers of different types of lymphocytes are thus likely to be associated with impaired tumor immunity, resulting in tumor progression (22, 23). CEA is elevated in peripheral blood in malignant tumors, especially in digestive tract tumors.

Serum CEA mainly reflects the tumor status, and PNI reflects the patient's overall condition, including nutritional and immune status. Their combination might be superior to either CEA or PNI alone for predicting the prognosis of patients with colon cancer. However, previous studies only focused on the prognostic association of a single variable with colorectal cancer alone. Research comparing different combinations of CEA and PNI in the prognosis of colon cancer remains lacking.

## Methods

### Patient Population

Databases from the Affiliated Cancer Hospital (learning set) and First Affiliated Hospital of Guangxi Medical University (validation set) were retrospectively reviewed. Approvals for the study were obtained from the two institutional review boards. The following patient clinicopathologic characteristics were obtained: gender, age, tumor location and size, operation mode (open vs. laparoscopic), preoperative blood test (CEA, CA199, albumin, and peripheral lymphocyte count), pathologic stage (T or N stage), differentiation degree of tumor cell, vascular or peri-neural infiltration, postoperative chemotherapy and information on deaths. Patients with stage II–III colon adenocarcinoma who received curative surgical excision from 2009 to 2019 at two medical centers were eligible. Exclusion criteria were as follows: (1) Younger than 18 years; (2) Preoperative neoadjuvant chemotherapy; (3) Palliative resection; (4) Multiple primary cancers; (5) Surgical history; (6) Incomplete preoperative laboratory data; (7) emergency surgery; and (8). incomplete follow-up information. The final learning and validation sets comprised 413 patients and 100 patients, retrospectively. The patients were started on semi-liquid to full liquid diet on admission. At 10 h before the operation, all patients were required to have oral sulfate-free polyethylene glycol electrolyte powder with 3,000 ml of water to remove all fecal contents.

### Calculation of Laboratory Data

Peripheral venous blood samples (10 ml) were collected from patients under empty stomach conditions in the morning. Serum albumin, CEA, and peripheral lymphocyte count were tested within 3 days before surgery. PNI was calculated as follows: PNI = serum albumin level (g/L) + 5 × total lymphocyte count (/L) (24).

### Model Construction and Internal Validation

Base on the results of multivariate analyses (*P* < 0.05), a nomogram was constructed by the combination of CEA/PNI with several other variables. External validation was applied to the validation set by the discriminatory power estimated by C-index and calibration curve.

### Statistical Analysis

Statistical analysis was performed using SPSS 26.0 software (IBM SPSS 26.0, SPSS Inc.) and R software 3.2.5. Pearson correlation analysis was conducted to evaluate correlations between absolute lymphocyte count and basal serum albumin. After descriptive analysis, the association of PNI with all clinicopathological variables was tested. Chi-square test was used to analyze categorical data. *T*-test was utilized to analyze continuous data. Any probability of 0.05 or less was considered statistically significant. The Cox proportional hazard model was employed to determine the independent factors that influence OS based on the variables from the univariate analyses. Variable association with OS was analyzed using the Kaplan–Meier method, and differences were tested with the log-rank test. A *P*-value of < 0.05 was defined as significant in the univariate and multivariate analysis of prognostic factors for OS. The optimal diagnostic cut-off value of the total number of lymphocytes, ALB, PNI, and tumor size was determined by calculating the Youden index of the ROC curve.

## Results

### Descriptive Statistics

**Figure 1:**
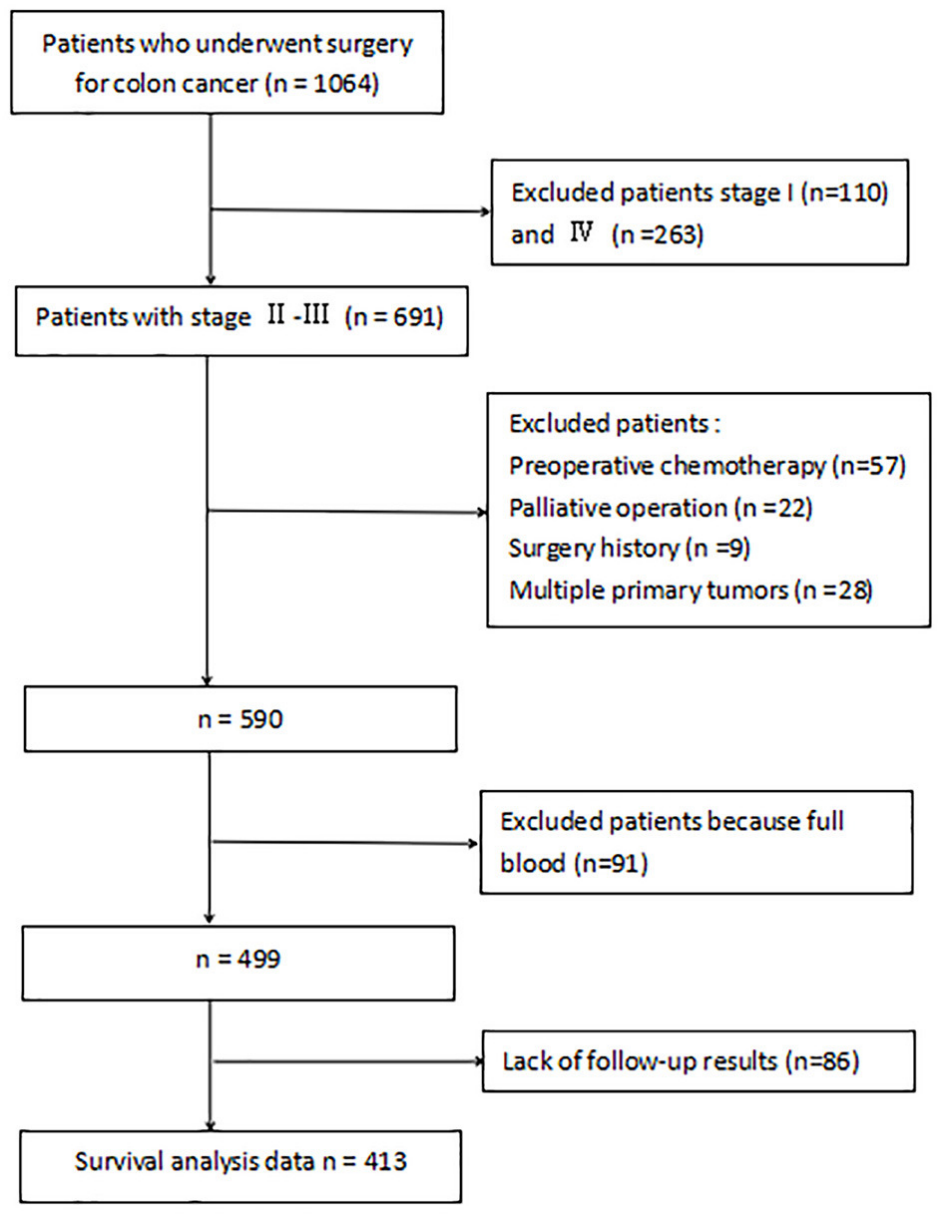
The data screening workflow.

**Figure 2:**
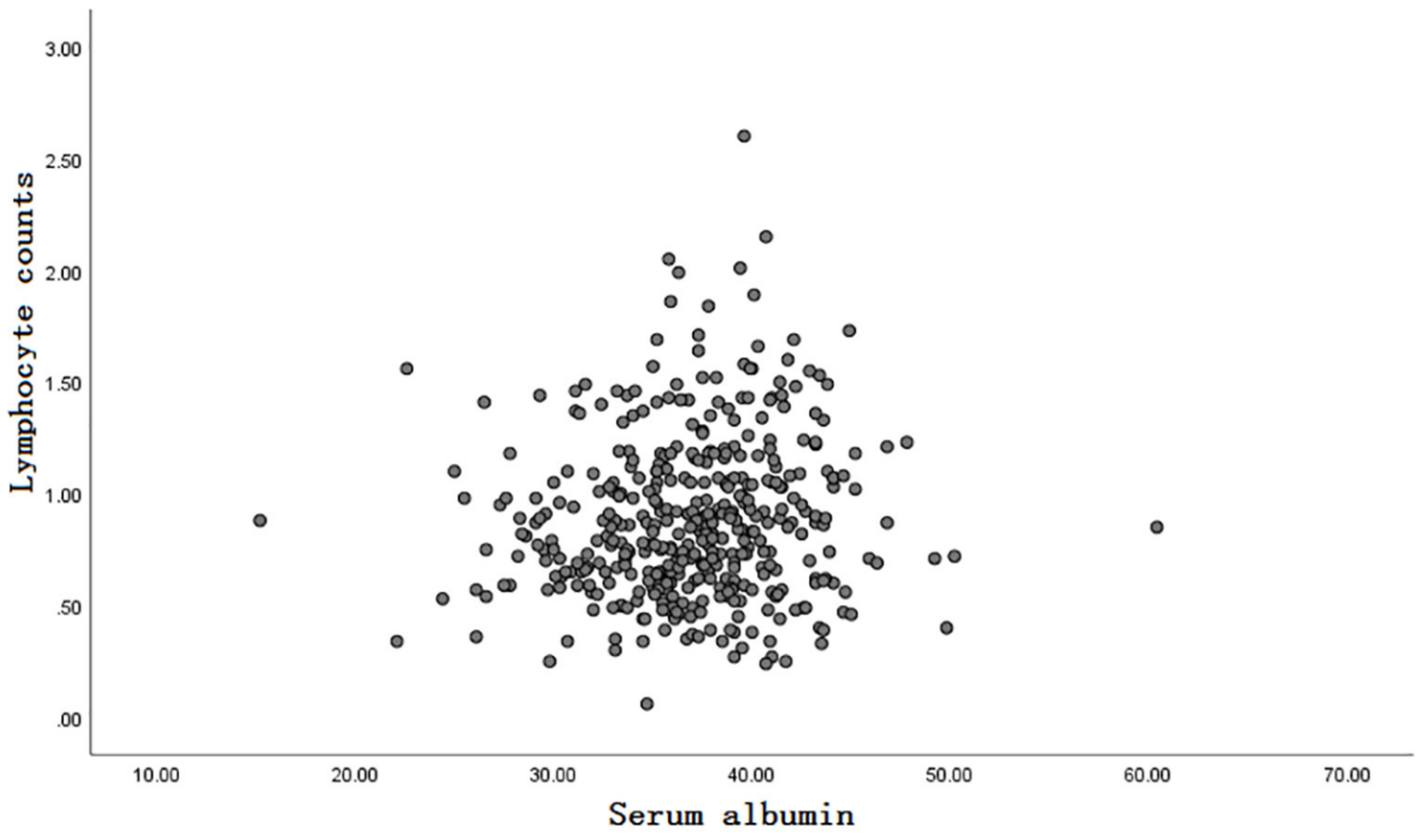
The correlation between absolute lymphocyte count and basal serum albumin.

**Figure 3:**
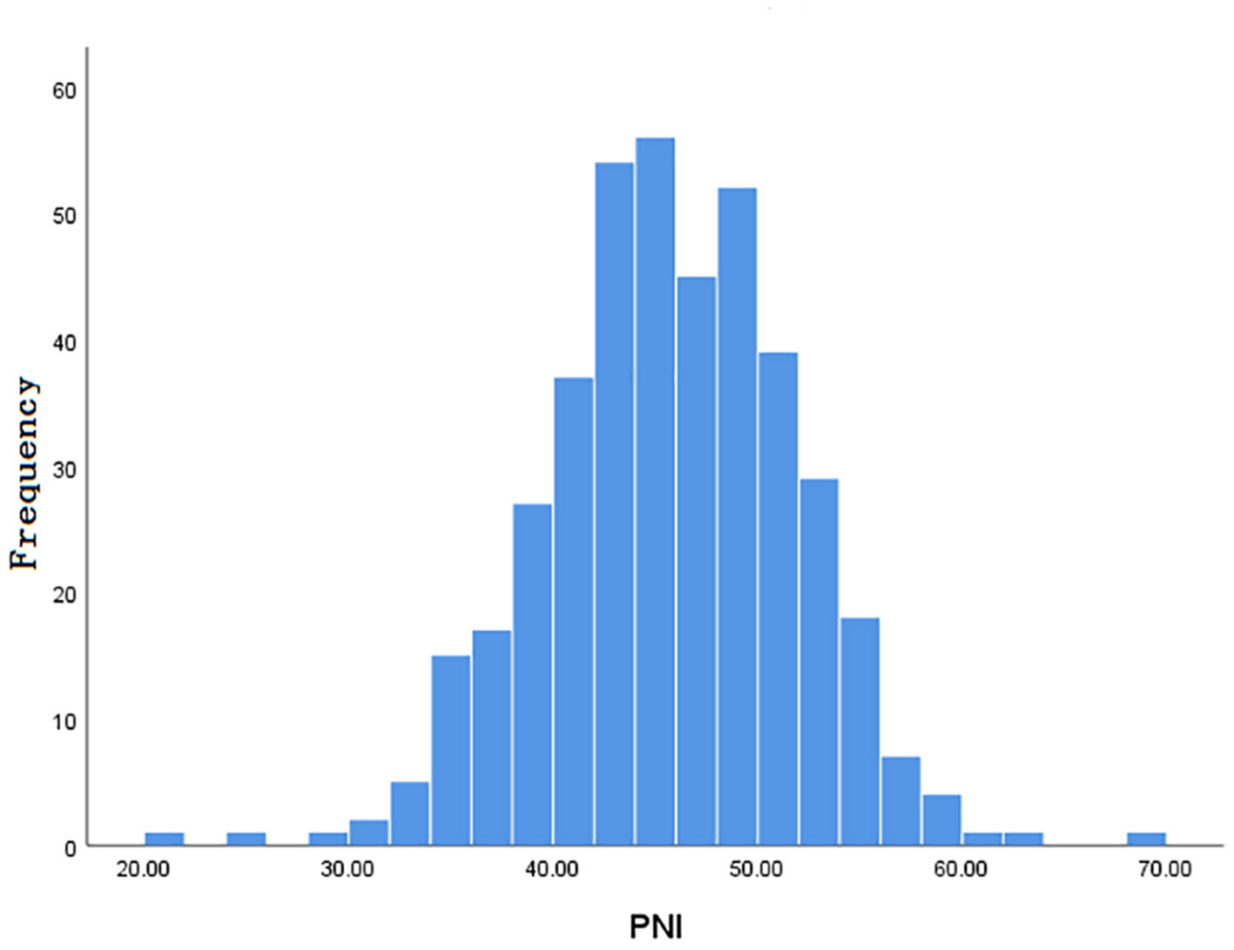
Normal distribution of PNI.

Inclusion and exclusion criteria are listed in Figure 1. A total of 413 patients were eligible for the learning set. The absolute lymphocyte count and basal serum albumin presented a poor correlation (*r* = 0.09; *P* = 0.068) (Figure 2). Mean PNI was 45 (SD 6.09; range 21.65–68.65), and its frequency distribution was normal (skewness −0.153, standard error [SE] 0.120; kurtosis 0.648, SE 0.240) (Figure 3).

An optimal cut-off of PNI (45) was identified by calculating the Youden index and was used to categorize patients into two groups: PNI ^high^ (*n* = 180) and PNI ^low^ (*n* = 233). Table 1 shows the relation between PNI and the clinicopathological parameters of patients. PNI^low^ was highly common in patients with large tumors (≧5 cm), right colon and lymph node metastasis (*P* < 0.05). The mean serum CEA level was 15.4 ng/ml (range: 0.2–1500 ng/ml). The patients were also classified into two groups according to serum CEA concentration as follows: CEA^low^(<5 ng/ml; *n* = 248) and CEA ^high^ (≧5 ng/ml; *n* = 165). The prognostic significance of the combination of CEA and PNI (CEA/PNI) was determined by grouping the patients into four as follows: CEA^low^/PNI ^high^ (*n* = 152), CEA ^low^/NI ^low^ (*n* = 96), CEA ^high^/PNI ^high^ (*n* = 81) and CEA ^high^/PNI ^low^ (*n* = 84).

**Table 1:** Relationships between preoperative prognostic nutritional index (PNI) and clinicopathological variables in leaning set with stage II–III colon cancer.

**Variable**	**PNI ^**high**^(*n* = 233)**	**PNI ^**low**^(*n* = 180)**	***P*-values**
Age (years, mean ± SD)	57.6 ± 0.8	57.9 ± 0.9	0.697
Gender			0.738
Male	148	112	
Female	85	68	
Tumor size (cm)			0.000
<5	140	61	
≧5	93	119	
Tumor location			0.011
Left	125	74	
Right	108	106	
Stage			0.036
II	113	106	
III	120	74	
Histology			0.233
Low	34	28	
Moderate	188	149	
High	11	3	
Depth of invasion			0.370
T1/2	12	6	
T3/4	221	174	
Lymph node metastasis			0.008
Absent	127	121	
Present	106	59	
Peripheral nerve invasion			0.119
Absent	96	88	
Present	137	92	
Vascular invasion			0.766
Absent	156	118	
Present	77	62	
CEA			0.014
Low (<5 ng/ml)	152	96	
High (≧5 ng/ml)	81	84	
CA199			
Low (<40 ku/L)	156	192	0.232
High (≧40 ku/L)	24	41	
Albumin			0.000
Low (<40 g/L)	109	178	
High (≧40 g/L)	124	2	
Blood lymphocytes			0.000
Low (<1,445/l)	134	101	
High (≧1,445/l)	139	79	

### Survival Analysis

**Figure 4:**
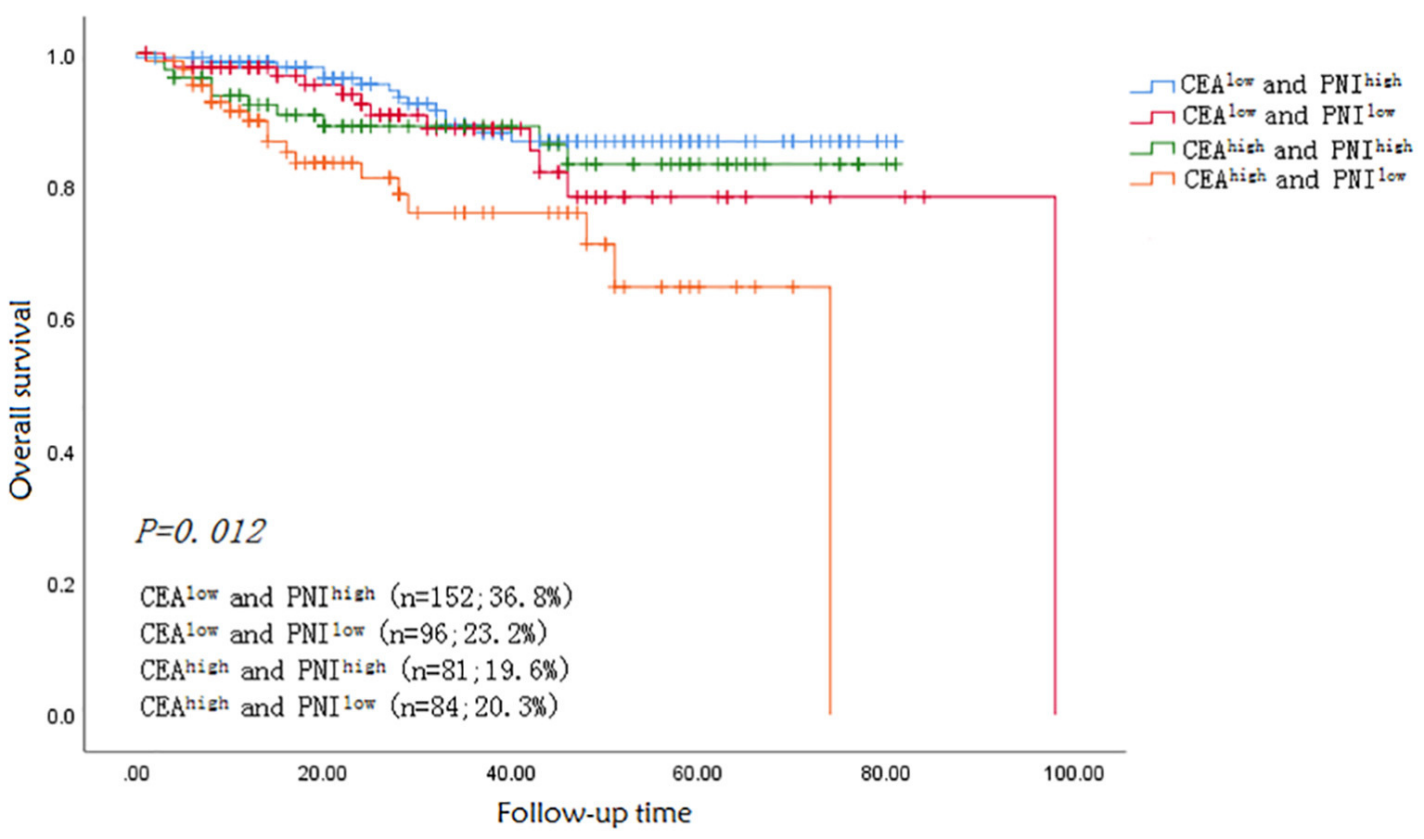
Kaplan–Meier (K–M) overall survival curve for CEA/PNI.

The median follow-up time was 33.6 months. Fifty-four patients died during the follow-up period from all causes. The 1-, 3- and 5-year OS rates were significantly higher in patients in the CEA^low^ (98, 88, and 75%) than in the CEA ^high^ (91, 82, and 66%) group (*p* = 0.012). The OS rates of patients with CEA ^low^/PNI ^high^, CEA ^low^/PNI ^low^, CEA ^high^/PNI ^high^ and CEA ^high^/PNI ^low^ were 98.5, 97.5, 92.6, and 89.5% for 1-year, respectively, and 88.6, 87.4, 86, and 74% for 3-year, respectively (*P* < 0.005). Kaplan–Meier survival curve for OS in patients with colon cancer was stratified by CEA/PNI (Figure 4) (*P* = 0.005). Survival status was determined by ROC curves and compared using the AUCs. The AUCs of CEA, PNI, and CEA/PNI for OS were 0.568, 0.427, and 0.797, respectively, indicating that CEA/PNI was more useful than either indicator alone for predicting OS in patients with colon cancer. Multivariate analysis identified CEA/PNI as an independent prognostic indicator in patients with colon cancer. Lymph node metastasis, peripheral nerve invasion, operation mode, and postoperative chemotherapy were also noted as indicators (Table 2).

**Table 2:** Univariate and Multivariate analyses of prognostic factors for overall survival in the learning set with stage II–III colon cancer.

**Variable**	**Univariate analysis**		**Multivariate analysis**	
	**HR (95% CI)**	***P*-value**	**HR (95% CI)**	***P*-value**
Age (years, mean ± SD)	0.645 (0.534–1.160)	0.562		
**Gender**				
Male/(female)	1.128 (0.674–1.979)	0.673		
**Tumor size (cm)**				
<5/(≧5)	1.299 (0.756–2.231)	0.344		
**Tumor location**				
Left/(right)	0.979 (0.571–1.680)	0.939		
**Stage**				
II/(III)	0.293(0.161–0.533)	0.000		
**Histology**				
Low/(high)	1.264 (0.283–5.655)	0.759		
Moderate/(high)	0.892 (0.215–3.698)	0.875		
**Depth of invasion**				
T1/2/(T3/4)	1.073 (0.334–3.445)	0.905		
**Lymph node metastasis**				
Absent/(present)	0.291 (0.164–0.516)	0.000	0.255 (0.137–0.472)	0.000
**Peripheral nerve invasion**				
Absent/(present)	0.395 (0.211–0.740)	0.004	0.488 (0.254–0.938)	0.031
**Vascular invasion**				
Absent/(Present)	0.381 (0.221–0.655)	0.000	–	–
**CEA**				
Low/(high)	0.509 (0.297–0.874)	0.014	–	–
**CA199**				
Low/(high)	0.479 (0.260–0.882)	0.018	–	–
**Albumin**				
Low/(high)	2.342 (1.200–4.572)	0.013	–	–
**Blood lymphocytes**				
Low/(high)	1.680 (0.974–2.895)	0.062		
**PNI**				
Low/(high)	1.924 (1.117-3.315)	0.018	–	–
**CEA/PNI**				
CEA^low/^PNI^high^/(CEA^high/^PNI^low^)	0.306 (0.152-0.618)	0.001	0.297 (0.142–0.624)	0.001
CEA^low/^PNI^low^/(CEA^high/^PNI^low^)	0.452 (0.213–0.958)	0.038	0.355 (0.169–0.790)	0.010
CEA^high/^PNI^high^/(CEA^high/^PNI^low^)	0.457 (0.210–0.994)	0.048	0.343 (0.153–0.767)	0.009
**Operation mode**				
Open/(Laparoscopic)	2.298 (1.332–3.964)	0.003	2.326 (1.325–4.083)	0.003
**Postoperative chemotherapy**				
No/(yes)	2.007 (1.136–3.544)	0.016	2.255 (1.235–4.117)	0.008

### Model Construction and External Validation

**Figure 5:**
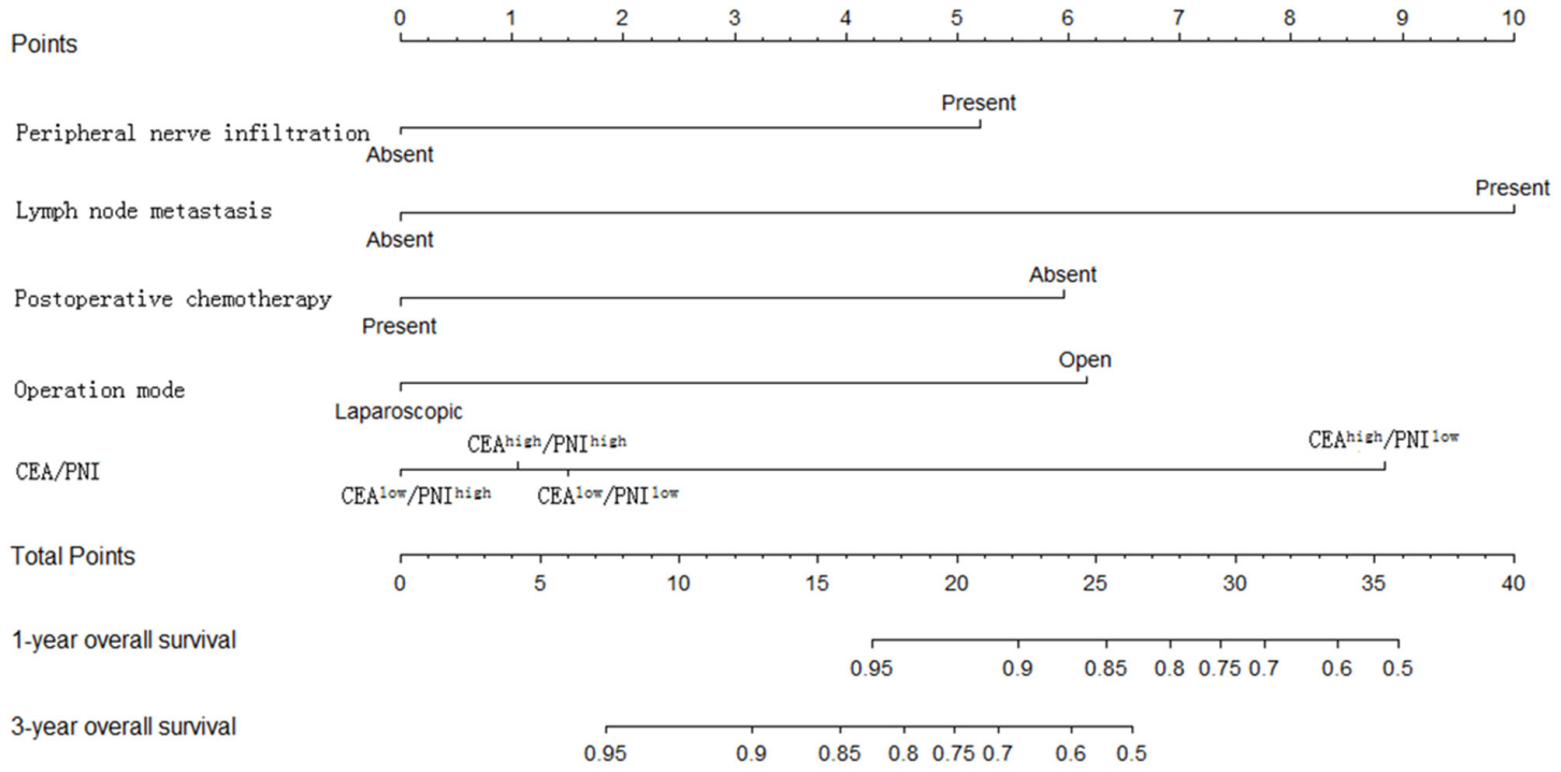
Nomogram predicting 1- and 3-year overall survival of colon cancer patients.

**Figure 6:**
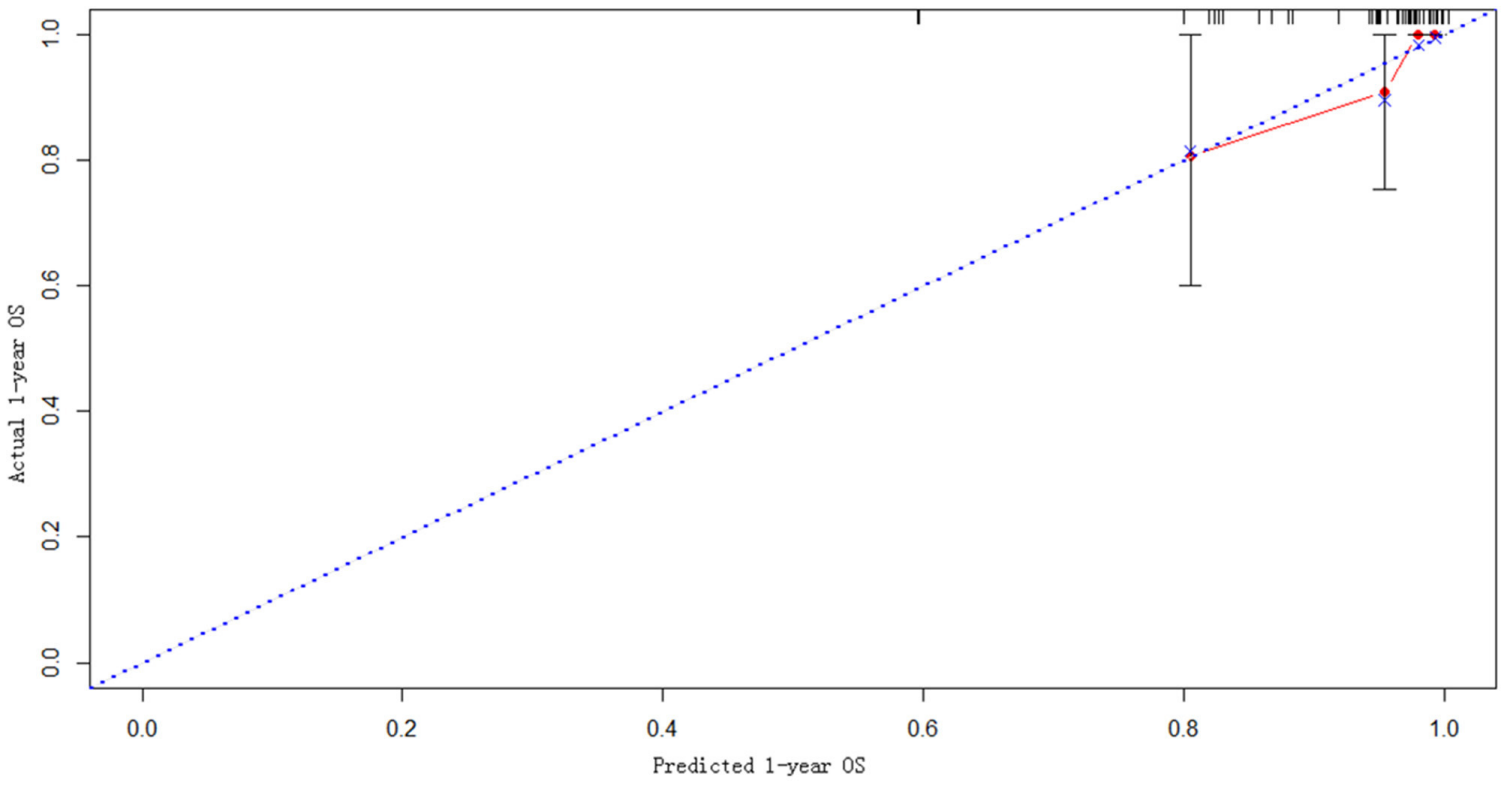
Calibration curves for 1-year prediction in the validation set.

**Figure 7:**
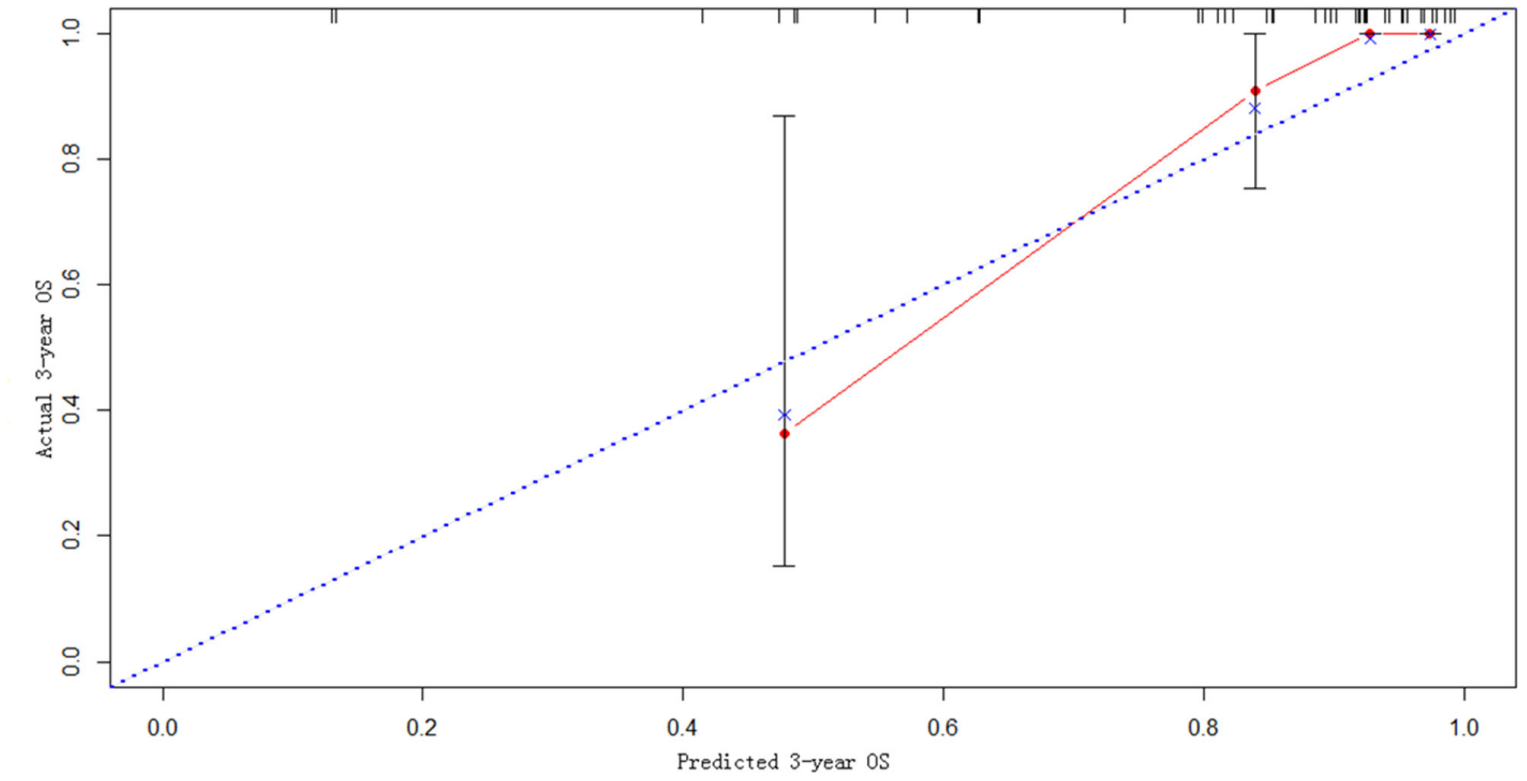
Calibration curves for 3-year prediction in the validation set.

The statistically significant clinicopathological variables found in the cohort of 413 patients in the learning set were used to develop a nomogram that predicts OS after curative resection (Figure 5). The validation dataset comprised 100 patients. The C-index for OS after curative resection was 0.788 in the learning set and 0.836 in the validation set. Calibration curves showed that the prediction curves coincided with the diagonal lines (Figures 6, 7).

## Discussion

An important association was found between CEA/PNI and OS in a single-center cohort of patients with stage II–III colon cancer. The results revealed that preoperative CEA/PNI is a prognosis-associated marker for patients with colon cancer. To the best of author's knowledge, this study takes the lead in evaluating the prognostic significance of preoperative CEA collaborated with PNI in patients with stage II–III colon cancer. Their combination was considered superior to either CEA or PNI alone to predict the prognosis of patients with colon cancer. Univariate analysis showed that factors affecting the prognosis of colon cancer included CEA, lymph node metastasis, peripheral nerve invasion, vascular invasion, albumin, peripheral blood lymphocytes, CEA/PNI, operation mode and postoperative chemotherapy. The final multivariate model strongly suggests that CEA/PNI, lymph node metastasis, CEA, peri-nerve invasion, operation mode and postoperative chemotherapy are independent prognostic variables.

Consistent with previous reports (6, 16), PNI was positively associated with the prognosis of colon cancer, indicating the values of immune and nutritional status as prognostic indicators. However, the mechanism by which PNI affects prognosis is unknown. PNI includes the measures of serum albumin and peripheral blood lymphocytes. Albumin, which constitutes up to 60% of plasma, is produced in the liver, reflects the nutritional status, is regulated by inflammatory cytokines and may play crucial roles in tumorigenesis and cancer progression (25). Serum albumin is positively correlated with the prognosis of colorectal cancer (26). Lymphocytes including NK cells, NKT cells, CD4+ T, and CD8+ T cells and B-cells are closely related to tumor immunity. Accumulating evidence implies that the decreased numbers of lymphocytes are associated with unfavorable prognosis in various cancers (27, 28). Several studies emphased that a cut-off value of PNI of 44 or higher is associated with a long OS on colorectal cancer (14, 29), and this result is highly consistent with our findings. Given that PNI is a continuous variable, the ultimate result may be uncertain in the process of conversion to the classification variable (14).

Preoperative CEA lacks sensitivity and specificity in disease diagnosis (30, 31) but is commonly used to assess the prognosis. The prognostic role of preoperative CEA concentration in early-stage CRC is controversial. This study revealed that preoperative CEA level is inversely correlated with the prognosis of patients with non-metastatic colon cancer. Multivariate regression analysis revealed that this factor is not an independent prognostic factor. For patients with early colon cancer, postoperative CEA levels can be used for predicting outcomes (32, 33). Studies from Asia suggested that postoperative CEA concentration ≥5 ng/ml is an important factor for poor prognosis in patients with stage II colorectal cancer. Preoperative high CEA concentration is a useful marker in follow-up, especially for stage II–III colon cancer patients. Further research suggested that elevated preoperative CEA level (≥5 ng/ml) loses its informative value when postoperative CEA level is normal (33). Although preoperative CEA may not be an independent prognosis factor for patients with stage II–III diseases (33, 34), it has predictive value in advanced/metastatic colon cancer (35) and can be combined with other markers or examination methods to evaluate the prognosis of early colon cancer (36).

Predictive models are particularly important for prognostic judgment and patient-physician clinical decision-making. This study used multivariate factors to develop a novel nomogram that is well-calibrated and externally validated. Subsequent verification revealed that the C-index reached 0.836, and the predicted value was highly consistent with the observed value in calibration curves.

However, this study has several limitations. The main pitfall is the retrospective design, second is the lack of validation data sets with sufficient samples, third is the unclear optimal variables cut-off value and the last is the small number of included patients. Therefore, multi-center and randomized control trials are needed to confirm the results. The main strength of the study is the prolonged follow-up periods for most survivors. Additionally, CEA, serum albumin and lymphocyte counts are inexpensive and easily obtained in any hospital.

In conclusion, multivariate regression analysis revealed that compared with CEA or PNI alone, the combination of CEA/PNI might provide relatively satisfactory prognostic information for patients with colon cancer. The presented prognostic model is inexpensive and can be easily constructed in clinical work.

## Conclusion

The combination of CEA and PNI is an independent prognostic factor, and a model based on this factor may be helpful in predicting the prognosis of patients with stage II–III colon cancer.

## Data Availability Statement

The raw data supporting the conclusions of this article will be made available by the authors, without undue reservation.

## Ethics Statement

The studies involving human participants were reviewed and approved by First Affiliated Hospital of Guangxi Medical University. The patients/participants provided their written informed consent to participate in this study.

## Author Contributions

Y-sX, GL, and CZ contributed equally to this study, performed the experiments, analyzed the data, and wrote the manuscript. Y-sX designed the experiments. H-gZ and S-lL collected the data. YC and L-xH checked and revised the manuscript. ZL confirmed all the data in the manuscript. C-yL and YC firstly modified the language of the manuscript. All authors read and approved the final manuscript.

## Conflict of Interest

The authors declare that the research was conducted in the absence of any commercial or financial relationships that could be construed as a potential conflict of interest.
